# Cognitive and emotional empathy in acute and remitted anorexia nervosa: a systematic review

**DOI:** 10.3389/fpsyt.2024.1385185

**Published:** 2024-05-24

**Authors:** Indigo E. Gray, Peter G. Enticott, Matthew Fuller-Tyszkiewicz, Melissa Kirkovski

**Affiliations:** ^1^ School of Psychology, Deakin University, Geelong, VIC, Australia; ^2^ Institute for Health and Sport, Victoria University, Melbourne, VIC, Australia

**Keywords:** anorexia nervosa, mentalising, empathy, acute, remission, state-trait

## Abstract

**Background:**

Impairments in empathy are well established in anorexia nervosa (AN). It is unclear, however, whether these deficits only occur in the acute phases of AN due to neurocognitive impacts of starvation (often referred to as context-dependent, or state-like), or if deficits remain once remission has been achieved (trait-like). This debate is commonly referred to as the ‘state vs trait’ debate.

**Objective:**

This systematic review aims to summarise existing literature regarding empathy in AN, and to investigate whether empathy deficits in AN are state- or trait-based.

**Method:**

A total of 1014 articles were identified, and seven articles remained after the screening process. These seven articles, comparing empathy across three groups (acute AN, remission of AN, and non-clinical controls), were evaluated and summarised in accordance with PRISMA guidelines. Articles were required to have included all three groups and report on either cognitive empathy and/or emotional empathy.

**Results:**

The majority of studies were of satisfactory quality. The results identified were inconsistent, with few articles lending some support to the ‘state’ hypothesis and others producing nonsignificant results.

**Conclusions:**

There is minimal literature comparing empathy in acute and remission phases of AN. While there were some inconsistencies in included articles, some data indicate that there may be slight improvements to emotional and cognitive empathy following recovery of AN. Further research is needed to better enrich knowledge regarding the role of state vs trait with regard to neurocognitive difficulties experienced by individuals with AN.

**Systematic review registration:**

https://www.crd.york.ac.uk/prospero/display_record.php?RecordID=335669, identifier CRD42022335669.

## 1 Introduction

Recent research has identified various neurocognitive impairments in individuals with anorexia nervosa (AN). From a social cognitive perspective, reduced empathy has been reported in AN samples (1–4), similar to effects observed in autism spectrum disorder (ASD) (5–7). Indeed, high levels of autistic traits, including socially relevant characteristics, have been noted to correlate with eating disorder psychopathology in clinical and non-clinical adolescent and adult samples (8–10). Notably, these empathy deficits have been shown to be restricted to AN specifically, and are not observed in other eating disorders such as bulimia nervosa (11).

Despite a growing body of evidence indicative of reduced empathic ability among AN samples, conflicting findings and inconsistencies are commonplace in the literature. Individuals with AN have difficulty identifying and recognizing the emotions of others (12, 13), which includes deciphering others’ intentions, thoughts, beliefs, and emotions (4). This skill is commonly termed *cognitive empathy*, and is also referred to as “mentalising” ability or “theory of mind”. Cognitive empathy is the capacity to understand and attribute emotional states to others by interpreting behaviour and intention, and is a crucial skill for developing and maintaining meaningful relationships (14, 15). By contrast, *emotional empathy* goes beyond inferring emotions and involves actual changes to an individual’s own emotional state, congruent with the emotions of the individual they are observing (16), and is also considered vital in establishing high quality interpersonal relationships (15). In this regard, Gaggero et al. (17) provide evidence to suggest that individuals with AN are able to appropriately adapt their facial expression in response to observing another individual in a distressing situation (17). Together, these findings are seemingly in line with the findings a review and meta-analysis which provides evidence supporting the notion of impaired cognitive empathy in AN, but also suggest that affective empathy might be relatively intact in among this population (18). In considering, however, that women with AN experience increased levels of alexithymia, or ability to recognize and label emotional states (19), the functional benefits of this “intact” emotional empathy is unclear. Other factors, such as participant age (18, 20) and methodological factors such as inconsistency between tasks and specific mentalising sub-domains measured (21) might account for some of the inconsistency observed in the literature.

Individuals with AN have been shown to have smaller social support groups (22), demonstrate diminished responsiveness and sensitivity to social cues (23, 24), and can be more negative in their interpretation of social situations (25), particularly when feeling ostracized (26). Research investigating the presence of social deficits in AN and illness outcomes found lower effectiveness of treatment and longer AN duration when social impairments were present (27–30). As these social and interpersonal impairments affect engagement in relationships, there is likely a secondary impact on intervention (31), as the typical approach of individual psychotherapies for AN often relies on quality therapeutic alliances (32). Indeed, McIntosh et al. (33) report interpersonal psychotherapy approaches to be least effective in treating AN. Interestingly, improvements in alexithymia symptoms has been observed to be related to improvements in eating disorder psychopathology (34), which highlights the importance of socio-cognitive awareness in the treatment of AN.

The consensus among researchers is that the presence of interpersonal difficulties in AN negatively impacts treatment outcomes (24, 35, 36). Given a large component of success in AN treatment is dependent on therapeutic relationship quality (37, 38), deficits in socialisation and possible poor relationship quality may hinder the therapeutic relationship and, by extension, the effectiveness of treatment.

While empathy impairments have been shown to be associated with AN (18, 39), they are not a diagnostic feature of the condition (40). Rather, such social difficulties are more typically associated with neurodevelopmental conditions such as ASD (40), clinically significant traits of which are highly prevalent in AN populations (41). Indeed, impairments in empathy similarly occur between autistic individuals and those with AN (12, 13, 42), though there is research to suggest greater difficulty among autistic cohorts (13). It is unclear, however, why individuals with AN display neurocognitive traits akin to ASD. A prominent theory explaining the presence of autistic features in AN speculates that the physiological and cognitive deficits caused by starvation can produce ASD-like symptoms, including empathy difficulties (20, 43, 44). This explanation is supported by research comparing acute versus recovered patients, which has identified that autistic symptomology is more profound in acute AN when starvation is present (also referred to as the ‘state-like’ hypothesis) (4, 45, 46). Conversely, it has also been suggested that autistic symptomology in AN is more stable or trait-based, where, irrespective of starvation and malnutrition effects, individuals in acute and recovered phases of AN both demonstrate cognitive and emotional empathy challenges (also referred to as the ‘trait-like’ hypothesis) (22, 24, 47, 48). Moreover, literature observing autistic symptomology prior to AN onset (49) also supports this “trait-like” hypothesis. Overall, a consensus remains unclear and these mixed findings, commonly referred to as the ‘state versus trait debate’, indicate a need for further investigation that directly compares the presence of ASD symptomology in acute AN and recovered AN to determine which hypothesis is supported or if elements of both ‘state’ and ‘trait’ may explain the presence of autistic traits in AN.

Given these gaps in the literature, this review aimed to summarise existing knowledge of empathy in AN to investigate the impact that starvation status may have on the presence of cognitive and emotional empathy deficits in AN, which will inform the ‘state vs trait’ debate. Such insights should have direct relevance for recommendations for treatment strategies.

## 2 Method

The review process was conducted in accordance with the PRISMA guidelines (50) and registered with PROSPERO (ID: CRD42022335669). We acknowledge here a slight deviation from the registered protocol. In the planning phases of this manuscript, it was intended that a systematic review and meta-analysis be conducted. As detailed below, however, only a small number of studies with varying outcome variables met inclusion criteria and were included for review. As such, only a systematic review is presented and there have been some changes to the team composition.

### 2.1 Search strategy

An initial literature search was conducted on the 12^th^ of August 2022 using the Medline, PsychINFO, and Scopus databases, which cover literature from psychological and other allied health disciplines. Search terms were (a) “Anorexia Nervosa” OR “Eating Disorder” OR “Anore*” and (b) “Mentalisation” OR “Mentali#ing” OR “Theory of Mind” OR “Mental flexib*” OR “Perspective Taking” OR “emotion* awar*” OR “emotion* perception” OR “emotion* detection” OR “emotion* identification” OR “emotion* recognition” OR “Cognitive Empathy” OR “Empathy” OR “Empathy Quotient” OR “Empath*” OR “EQ” OR “compassion*” OR “Emotion* Contagion.” Key words were selected based on the variables of interest for review, the author’s expertise, and by screening key words often presented in relevant literature. Searches in each database were limited to English language and peer-reviewed articles. This search was repeated on the 19^th^ of August 2023. One reviewer (IEG) completed searchers and compiled outcomes into the RAYAAN article screening platform (51). One additional paper that was not identified by the initial search strategy but fit the above specified criteria was included by the researchers. To check that any additional papers had not similarly been missed by the initial search, an expanded search was conducted in December 2023 using the following *additional* search terms: “Social Cognition” OR “Affective Cognition” OR “Reading the Minds in the Eyes” OR “The Films Expressions Task” OR “The Multifaceted Empathy Test” OR “The Frith-Happé Animations.” No additional papers were identified from this search.

#### 2.1.1 Eligibility criteria

Articles were considered for inclusion in the review if they met the following criteria:

1. Published in English.
2. Peer-reviewed.
3. Included three groups: an AN acute group, an AN remission whose weight had been restored, and a non-clinical comparison group (Healthy Control [HC] group). AN groups were required to confirm via self-report that they had received a diagnosis of AN according to either the Diagnostic and Statistical Manual of Mental Disorders or the International Classification of Diseases diagnostic criteria.
4. Reported on at least one of the outcomes of interest: cognitive empathy or emotional empathy. No restrictions were made on the types of outcome measures studies used to assess these outcomes.

No restrictions were placed on date of publication or the age of the sample.

### 2.2 Screening

**Figure 1:**
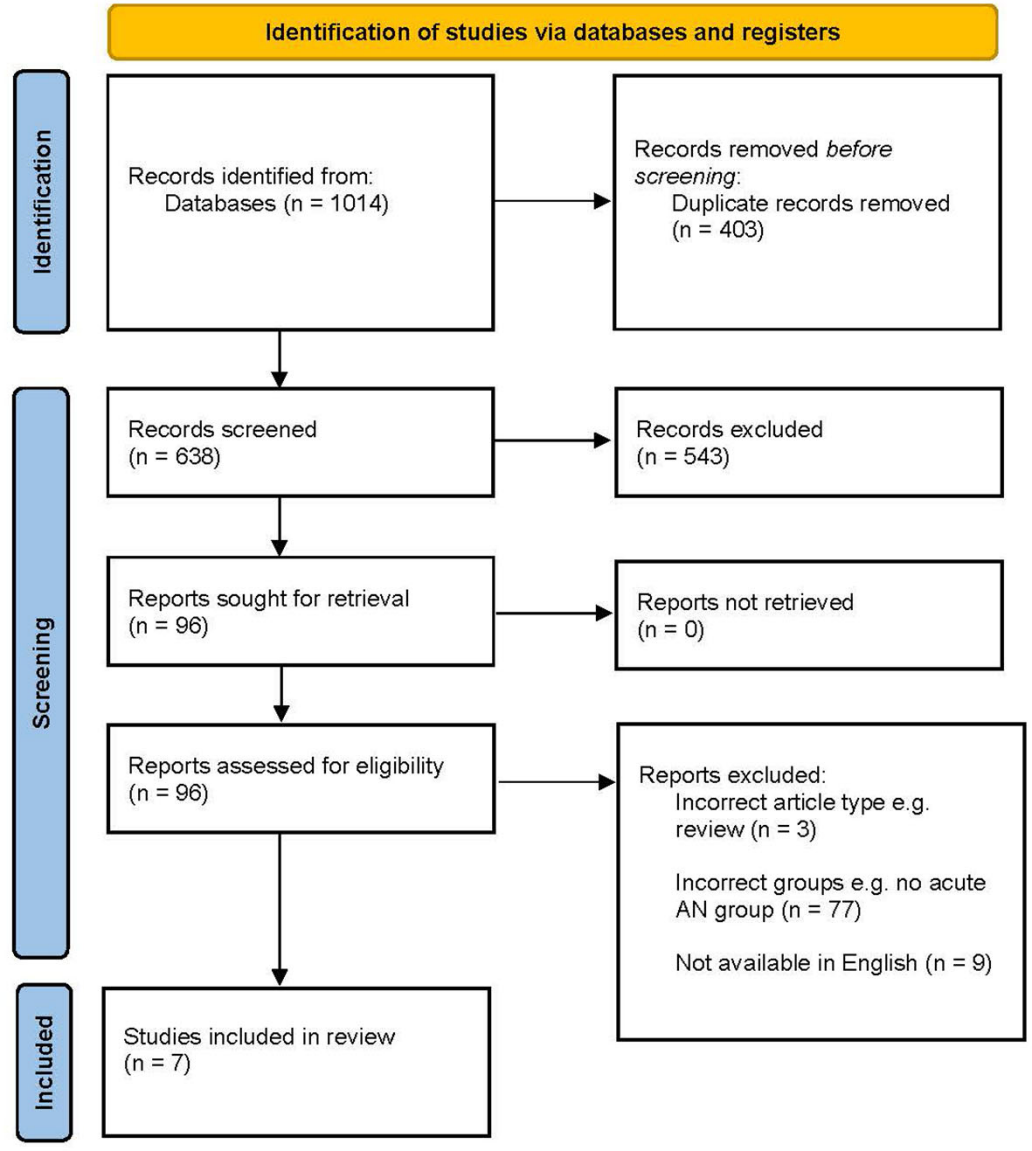
PRISMA flowchart describing identification of literature. Adapted from Page et al. (50).

As depicted in **Figure 1**, the initial search yielded 1014 articles. 403 duplicates were removed, and two reviewers (IEG and MK) then completed an independent and blind title and abstract screening of all 638 articles. Any conflicts from this screening were resolved through mutual reassessment of the articles. 6.4% of the 638 articles presented a conflict, and, after resolution, 96 articles remained to be screened as full reports. From this pool of articles, 6 met inclusion criteria and were thus included in the present review. No additional articles were found on the repeated search in August or December, 2023. However, one additional article was found by the researchers outside of the formal search process. As such, 7 articles are included in the review.

### 2.3 Data extraction

The following details were extracted from each included article: first author, year of publication, overall and subgroup sample size, age, body mass index (BMI), biological sex percentages of the sample, and outcome measures used. All data were manually extracted from each included article.

### 2.4 Quality assessment

The quality of included studies was assessed using the Newcastle-Ottawa Scale (NOS) (52), which evaluates studies in relation to three categories: participant selection (representativeness, size, non-respondents), comparison between groups in the study (ascertainment of screening, comparability of subjects), and outcome assessment (assessment, statistical test). There are four criteria for sample selection, two criteria for comparison, and three criteria for outcome assessment. The NOS uses a star-based system where each study can obtain one star for each assessed criterion in the selection and outcome criterion and a maximum of two stars for the comparability criterion, thereby achieving a maximum of nine stars overall. Studies are then determined to be either unsatisfactory (0-4 stars), satisfactory (5-6 stars), good (7-8 stars), or very good (9-10 stars) based on their star rating.

## 3 Results

### 3.1 Description of studies

A summary of these data is provided in **Table 1**. Of the seven included studies the average age range was between 18-30. Half of the studies had an all-female sample; however, all studies’ samples were above 90% female. AN diagnosis was determined using the DSM-5 in three studies (55–57), using the DSM-IV-TR in the three studies (14, 54, 58), and using the ICD-10 in one study (53). All seven articles employed quantitative data analysis approaches, but each investigates different outcome measures to assess empathy.

**Table 1:** Summary of studies.

Author and Year	Sample Size (subgroups)	Female %	Average Age (SD) of each subgroup	AN Criteria	BMI Subgroup Average (SD)	Outcome Assessed	Outcome Measure	Key Findings
53	112 (AN-A=43, AN-R = 28, HC=41)	AN-A = 100% AN-R = 100% HC= not provided	AN-A = 16.1 (1.5) AN-R = 18.4 (1.6) HC = 17.7 (2.2)	ICD-10	AN-A = 16.6 (1.2) AN-R = 21.3 (1.8) HC = 22 (2.6)	Cognitive Empathy	The Reading the Mind in the Eyes Task, Mini-profile of Nonverbal Sensitivity (MiniPONS), The Animated Triangles Task, and The Awareness of Social Inference Test	No significant differences were observed on the Animated Triangles Task (p = .24) or the Awareness of Social Inference Test (p = .15). On the MiniPONS, the AN-A group performed significantly better than the HC group to a medium effect (p = .05, d = 0.5). The AN-A group performed significantly better than the AN-R group to a large degree (p = .008, d = 0.8). No differences were found between the AN-R and the HC group. On the Reading the Mind in the Eyes Task, although there was an overall difference between the groups (p = .04) no significant differences were found between the three groups in *post hoc* analyses.
54	175 (AN-A=50, AN-R=35, HC=90)	All groups = 100%	AN-A = 26.7 (9.82) AN-R = 29 (10.62) HC= 28.50 (9.93)	DSM-IV	AN-A = 15.38 (1.83) AN-R = 21.15 (1.76) HC= 21.61 (1.89)	Cognitive Empathy	Pictorial Stroop Task and The Reading the Mind in the Eyes Task	Both AN-A and AN-R groups performed significantly worse than HC with a small effect size (d = 0.25, d = 0.27 respectively). There was no significant difference between AN-R and AN-A and had a negligible effect size (d = 0.09).
55	140 (AN-A=45, AN-R = 49, HC=46)	AN-A = 93.5% AN-R = 98% HC= 91.1%	AN-A = 27.04 (8.92) AN-R = 26.00 (8.10) HC = 23.87 (4.52)	DSM-V	AN-A = 15.75 (1.41) AN-R = 21.12 (1.91) HC = 21.69 (1.88)	Cognitive Empathy	The films Expressions Task (FET) -cognitive empathy	No significant difference between groups was found, however, there was a small effect size (η_p_^2^ = .02).
56	147 (AN-A=51, AN-R =50, HC=46)	AN-A = 92.2% AN-R = 98% HC= 93.5%	AN-A=27.57 (8.52) R= 26.33 (8.04) HC= 24.37 (4.43)	DSM-V	AN-A= 15.72 (1.41) AN-R = 21.20 (1.95) HC= 21.69 (1.88)	Cognitive and Emotional Empathy	The Multifaceted Empathy Test (MET) and The Mini-profile of Nonverbal Sensitivity (MiniPONS)	No significant differences were found between groups on either outcome and reported effect sizes were small for cognitive empathy (F = 0.72, p = .49, η_p_^2^ = .01), and small for emotional empathy (F = 1.65, p = .20, η_p_^2^ = .02). However, reported *post-hoc* tests indicated a trend towards AN-A scoring lower than AN-R (p = .057) with a small derived effect size of (d = 0.36). In addition, emotional empathy and cognitive empathy were both significantly positively correlated with BMI with a small effect size (r = .17, p = .04).
57	188 (AN-A=67, AN-R =49, HC=70)	All groups = 100%	AN-A= 18.70 (2.78) AN-R =19.72 (3.27) HC = 19.64 (3.30)	DSM-V	AN-A=16.61 (1.41) AN-R = 20.84 (2.26) HC= 22.82 (3.31)	Cognitive Empathy	The Frith-Happé animations	No significant differences between groups and no trends.
58	107 (AN-A=28, AN-R =25, HC=54)	All groups = 100%	AN-A= 26.3 (7.9) AN-R = 29.5 (9.2) HC= 29.4 (9.6)	DSM-IV	AN-A= 15.5 (1.3) acute AN-R= 20.1 (1.9) HC= 23.1 (3.9)	Emotional Empathy	Socio-Emotional Questionnaire (SEQ)	There was a significant difference between the groups on empathy (F = 3.11, p = .43). AN-A performed significantly worse than HC (p <.05) with a medium derived effect size (d = 0.66). In addition, there was a significant medium positive correlation between BMI and emotional empathy (r = .29, p = .003).
14	111 (AN-A= 40, AN-R =24, HC=47)	AN-A=92.5%, AN-R =95.8%, HC=78.72%	AN-A = 27.3 (10) AN-R = 29.9 (7.7) HC = 29.8 (8.0)	DSM-IV	AN-A=16.6 (1.3) AN-R = 20.8 (2) HC= 23 (2.8)	Cognitive Empathy	Reading the Mind in the Eyes Task and the Level of Emotional Awareness Scale (LEAS)	AN-A performed significantly worse compared to AN-R (d = -0.77, p = .03). and HC (d = -0.85, p <.001) when reading negative emotions, with both comparisons demonstrating large effect sizes. The AN-R group performed comparably the HC group. For positive emotions, AN-A performed the worst, then the AN-R and then HC but no significant differences were found between the groups. However, there was a small effect size when AN-R was compared to AN-A (d = 0.29) and when AN-R was compared to HC (d = -0.27). For neutral states, there were no significant differences, but the AN-A group demonstrated the worse performance, followed by the HC group and then the AN-R group who performed best.

AN-A, Acute AN Group; AN-R, Remission AN Group; HC, Healthy Control Group; BMI, Body Mass Index; SD, Standard Deviation; d, Cohen’s d; η_p_^2^, Partial eta squared; r, correlation coefficient.

### 3.2 Quality assessment

As seen in **Table 2**, only two studies received a ‘good’ rating. All other studies were ‘satisfactory’, and one study was ‘unsatisfactory’. All studies had representative samples (employed random sampling), used validated measures to confirm AN diagnosis and selected appropriate statistical tests to analyse data. The majority of studies had sound response rates from participants with few dropouts. However, only two studies explicitly investigated and accounted for potential confounds such as gender and comorbidities, e.g. anxiety, depression and ASD, and only one study included a justification for their sample size by providing a power analysis. All studies failed to either report or utilise blinded assessment.

**Table 2:** Newcastle-Ottawa Scale Quality Assessment: Star Totals for Each Domain.

	Selection				Comparability	Outcome		Overall Grade
Paper	Representativeness	Size	Non-respondents	Ascertainment of screening	Comparability of subjects	Assessment	Statistical test	
53	*		*	**	**		*	G
54	*			**			*	U
55	*		*	**			*	S
56	*		*	**			*	S
57	*	*		**			*	S
58	*		*	**			*	S
14	*		*	**	**		*	G

* = one point; ** = two points. U, Unsatisfactory (0-4 stars); S, Satisfactory (5-6 stars); G, Good (7-8 stars).

### 3.3 Emotional empathy

Two articles investigated emotional empathy (56, 58). While the results of both studies indicated superior performance on emotional empathy tasks in control participants, the small effect only reached statistical significance in one of these studies (56) Both studies also indicated that the AN remission group performed better than the AN acute group but worse than the non-clinical comparison group, though the effects were small and not significant. Additionally, both articles observed a significant positive correlation between BMI and emotional empathy, further supporting that emotional empathy improves as weight increases. Moreover, 58 also noted a significant difference between the AN acute group and the non-clinical comparison group. As both studies reported worse performance by the AN groups, this outcome confirms emotional empathy deficits in AN observed in previous literature (1–4).

### 3.4 Cognitive empathy

One study found that the AN remission group performed significantly better than the AN acute group with a large effect size, and performed comparably to the health controls group when recognising negative emotional states (14). Additionally, there was no significant difference between groups for positive valance, but the data indicated that the AN acute group performed (non-significantly) the worst, followed by the AN remission and then health controls group, again with small effects. There was, however, a small effect size involving the AN remission group, whereby this group demonstrated superior performance to the acute AN group, and inferior performance to the healthy controls group (14). Once again, for neutral states, the same pattern of performance was observed where the AN acute group performed worst, albeit this was not statistically significant, followed by the AN remission and then the healthy controls group, which was again not statistically significant. Although not significant, four (14, 28, 55, 56) of the six articles investigating cognitive empathy observed that the healthy controls group demonstrated the highest accuracy in emotion recognition followed by AN remission group and then the AN acute group.

Moreover, one study (56) reported a small but significant positive correlation between BMI and cognitive empathy. Furthermore, one study discerned that the AN acute group performed significantly worse than the non-clinical comparison group, with a large effect size, once again confirming cognitive empathy deficits in AN in line with previous literature (14).

In addition, one study identified that both the remission and acute AN groups performed significantly worse than the non-clinical comparison group to a small degree, suggesting that even individuals with AN who have achieved weight restoration still may show impairments in theory of mind (54). Similarly, one study (53) found that the AN acute group performed better than the AN remission and HC groups on one outcome measure. However, these differences were not observed on any of the three other outcome measures. By contrast, Leslie et al. (57), did not observe any groups differences or trends in cognitive empathy outcomes.

## 4 Discussion

The primary aim of this review was to summarise the available literature investigating empathy in AN and to investigate the impact that starvation might have on the presence of cognitive and emotional deficits in AN. Given that, potentially domain-specific (18), reduced empathic ability is commonly reported among AN samples (18, 39), which likely impacts other areas of functioning such as socialisation (8, 9), the present review examined research investigating elements of both emotional and cognitive empathy. It is critical that researchers and clinicians better understand empathy deficits in AN populations and how they may impact socialisation and relationships in order to reduce any impacts on therapeutic relationships and subsequent intervention effectiveness.

From the small body of literature available, it can be concluded that while impairments in empathy may improve once remission of AN has been achieved, the effects are small and further research utilising more comprehensive assessment and larger sample sizes comparing the acute and remission phases of AN is critical. Nevertheless, the reviewed literature could be considered somewhat consistent with previous research highlighting starvation-induced psychological impairments can produce cognitive and socialisation difficulties, which might lend some insight as to why autistic trait are so commonly observed in AN populations (20, 43, 44, 59). However, as most studies reported nonsignificant results, conclusions that can be drawn are limited in this instance. While some patterns in the data indicated that the remission groups performed better than acute AN groups and worse than nonclinical control groups, only two of the seven studies reported observed this pattern at a statistically significant level. A possible explanation for this inconsistency is the difference in outcome measures selected in each included article. Almost all studies utilised different measures, which may assess different empathy domains and also vary in their sensitivity to capturing empathy deficits. Additionally, previous literature has typically compared participants in the acute stage of AN to control groups with no psychiatric history and consistently observed significant deficits in empathy in AN in this regard (39, 42, 60). Thus, the inclusion of remission groups is a comparatively new area of interest and, as such, the literature is scarce and lacks consistency.

Additionally, one study (57) did not support the state debate and, instead, identified comparable performance across all three groups. Unexpectedly, no differences were observed between the acute AN and nonclinical comparison groups on cognitive empathy skills, which is inconsistent with previous literature (2, 53, 61). This outlying study utilised the Frith-Happé animations which, unlike the other performance-based measures, presents animated triangles as opposed to faces to assess theory of mind. It is possible the differentiation in this presentation of the measure could explain why the findings were not consistent with previous literature.

### 4.1 Limitations and direction for future research

There are several limitations within this systematic review that are important to consider when interpreting findings. Firstly, only a small number of studies were obtained, particularly for those assessing emotional empathy, and used various measures. Studies reviewed were often underpowered, which was highlighted by the number of patterns in the data that did not reach significance.The diversity in measures used for each study presents an additional confound. One study (58) gathered self-report data, requiring participants to reflect on past experiences, while all other studies utilised performance-based measures to capture empathy levels at present. This made comparing results and drawing conclusions difficult and highlights the need for additional research in this area.

Additionally, the samples within each included study overwhelmingly represented female participants. While a diagnosis of AN is more common among biological females compared to males (40) and thus sample demographics are generally aligned with clinical expectations, AN does still occur in males and there is, therefore, a gap in our understanding of the male profile of AN and the consequent neurocognitive implications. As a result, cognitive and emotional empathy could not be sufficiently examined in males. Indeed, a possible area of exploration for future research could be to examine possible differences in empathy as a factor of biological sex and also of gender to determine if there are discrepancies. Indeed, there is a higher prevalence of AN among gender diverse populations (62) and is therefore important to consider in addition to biological sex. Any differences are likely beneficial to consider in treatment to ensure intervention effectiveness as empathy deficits may impact upon the therapeutic relationship.

Finally, a factor not explored yet in the literature is the time spent in recovery. Those in remission from AN whose weight has been clinically restored may demonstrate increasing improvements in their empathy skills as they continue to recover. Further suggestions for future research, therefore, relate to the comparison of empathy in acute AN and remission across the life span, between biological sexes and among gender diverse populations, and the longitudinal recovery of AN and how empathetic ability changes across this time span. Additionally, future research should consider investigating other areas of neurocognition which may be affected be starvation such as executive functioning and memory.

### 4.2 Summary and conclusion

While it is generally noted in the literature that people with AN present with some degree of potentially domain specific (18) reduced empathic ability, the limited literature considered in this present review provides incentive for deeper investigation of the role of starvation on neurocognitive performance, particularly in the socio-cognitive domain. However, understanding the underpinnings of why this impairment is present may be crucial in informing treatment decision making and altering ‘gold- standard’ intervention for a condition that is proven difficult to treat with long-term efficacy. The current patterns in the data are inconsistent, which could be attributable to the varying methodological and sample characteristics of each study. Additionally, studies predominantly reported non-significant outcomes. Our findings, therefore, suggest that while emotional empathy and cognitive empathy may improve throughout recovery, further investigation utilising comprehensive measures of empathy is necessary. It is anticipated that these findings will assist clinicians treating AN to account for these empathetic deficits when building a therapeutic relationship with clients from this population, setting client goals, and delivering interventions to enhance treatment outcomes.

## Data availability statement

The original contributions presented in the study are included in the article/supplementary material. Further inquiries can be directed to the corresponding author.

## Author contributions

IG: Conceptualization, Data curation, Formal analysis, Investigation, Methodology, Project administration, Writing – original draft, Writing – review & editing. PE: Resources, Supervision, Writing – review & editing. MF: Conceptualization, Supervision, Writing – review & editing. MK: Conceptualization, Formal analysis, Methodology, Project administration, Supervision, Writing – review & editing.
